# Bloom syndrome helicase contributes to germ line development and longevity in zebrafish

**DOI:** 10.1038/s41419-022-04815-8

**Published:** 2022-04-18

**Authors:** Tamás Annus, Dalma Müller, Bálint Jezsó, György Ullaga, Barnabás Németh, Gábor M. Harami, László Orbán, Mihály Kovács, Máté Varga

**Affiliations:** 1 Department of Genetics, ELTE Eötvös Loránd University, Budapest, Hungary; 2 Institute of Enzymology, Research Centre for Natural Sciences, Eötvös Loránd Research Network, Budapest, Hungary; 3 Department of Anatomy, Cell and Developmental Biology, ELTE Eötvös Loránd University, Budapest, Hungary; 4 ELTE-MTA „Momentum” Motor Enzymology Research Group, Department of Biochemistry, ELTE Eötvös Loránd University, Budapest, Hungary; 5 Frontline Fish Genomics Research Group, Department of Applied Fish Biology, Institute of Aquaculture and Environmental Safety, Hungarian University of Agriculture and Life Sciences, Georgikon Campus, Keszthely, Hungary; 6 MTA-ELTE Motor Pharmacology Research Group, Department of Biochemistry, ELTE Eötvös Loránd University, Budapest, Hungary

**Keywords:** Disease model, Germline development

## Abstract

RecQ helicases—also known as the “guardians of the genome”—play crucial roles in genome integrity maintenance through their involvement in various DNA metabolic pathways. Aside from being conserved from bacteria to vertebrates, their importance is also reflected in the fact that in humans impaired function of multiple RecQ helicase orthologs are known to cause severe sets of problems, including Bloom, Werner, or Rothmund-Thomson syndromes. Our aim was to create and characterize a zebrafish (*Danio rerio*) disease model for Bloom syndrome, a recessive autosomal disorder. In humans, this syndrome is characterized by short stature, skin rashes, reduced fertility, increased risk of carcinogenesis, and shortened life expectancy brought on by genomic instability. We show that zebrafish *blm* mutants recapitulate major hallmarks of the human disease, such as shortened lifespan and reduced fertility. Moreover, similarly to other factors involved in DNA repair, some functions of zebrafish Blm bear additional importance in germ line development, and consequently in sex differentiation. Unlike *fanc* genes and *rad51*, however, *blm* appears to affect its function independent of *tp53*. Therefore, our model will be a valuable tool for further understanding the developmental and molecular attributes of this rare disease, along with providing novel insights into the role of genome maintenance proteins in somatic DNA repair and fertility.

## Introduction

Bloom Syndrome (BSyn, OMIM #210900) is a rare monogenic autosomal recessive disorder [1, 2] with symptoms ranging from below average height and weight and lesions on exposed skin areas, to reduced fertility and shortened life expectancy most often brought on by heightened proneness to cancer development [3]. The defective gene (*BLM;* [4]) responsible for the condition was mapped to 15q26.1 in the human genome and encodes a 3′-5′ DNA helicase showing homology to the *E. coli* RecQ protein [5, 6].

RecQ homologs are evolutionarily conserved proteins involved in a variety of genome integrity maintenance mechanisms [7]. BLM itself is required for precise double-stranded DNA break (DSB) repair, crossover patterning regulation, telomere maintenance, processing of DNA replication intermediates, and rDNA metabolism [8–10].

**Fig. 1:**
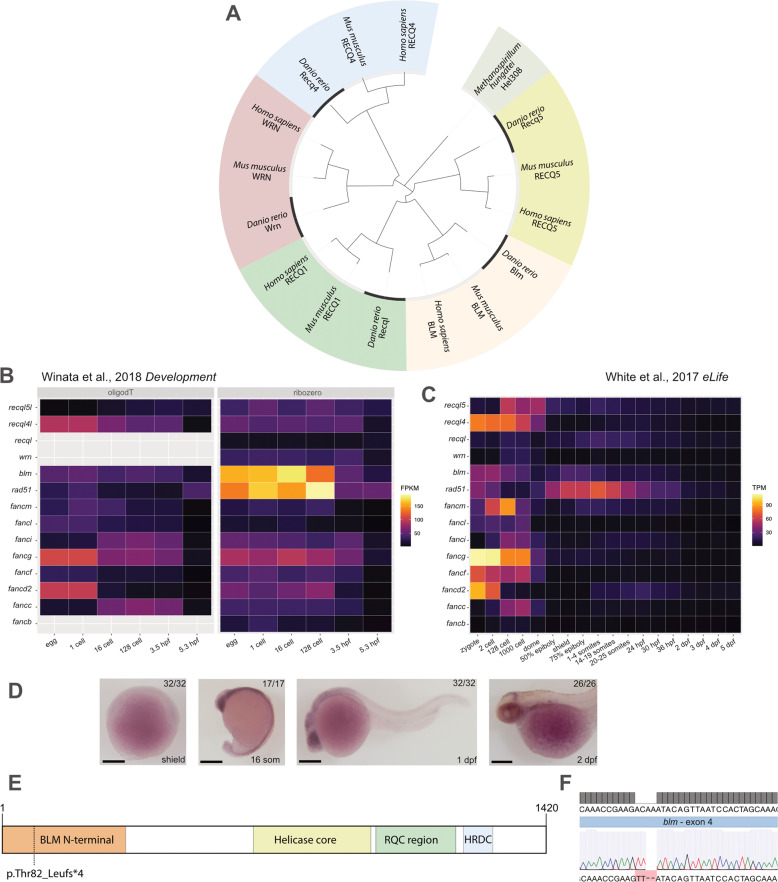
RecQ homologs in the zebrafish genome and their expression. **A** Phylogenetic relationships of the five RecQ homologs that can be identified in the zebrafish genome based on similarities between the helicase ATPase and helicase C-terminal domains. (*M. hungatei* Hel308 DNA helicase was used as an external reference for eukaryotic RecQs). **B**, **C** The expression of genes related to double-strand DNA breaks during zebrafish development. Fragments per kilobase of exon model per million reads mapped (FPKM) and transcripts per kilobase million (TPM) values of the different genes at given stages as shown by the datasets in the respective papers (White et al., 2017; Winata et al., 2018). **D** Spatial distribution of *blm* RNA during early stages of development detected by whole-mount in situ hybridization. The number of embryos showing the expression pattern is indicated in the upper right corner. (All lateral views; for shield and 16 somite stages embryos ventral is to the left and dorsal to the right, while for 1 and 2 dpf embryos anterior is to the left and posterior is to the right, respectively. Scale bars: 150 μm.). **E** Schematic domain structure of zebrafish Blm and the position of the p.Thr101Leufs*4 mutation. (RQC RecQ-C-terminal domain, HRDC helicase and RNaseD C-terminal.). **F** Sanger sequencing shows the presence of the c.301_304delACAAinsTT allele (*elu15*) in exon 4 of a *blm*^−*/−*^ embryo.

Zebrafish is an attractive candidate to model RecQ family-related diseases as all five human RecQ paralogs (*RECQL1, WRN, BLM, RECQL4*, and *RECQL5*) have a single zebrafish ortholog each (Fig. 1A). In order to create a novel model for BSyn, we generated a null allele for *blm* and analysed its potential effects on lifespan, gonad differentiation, fertility, histology, and DNA repair efficiency.

Multiple genes involved in DSB repair can influence sex determination (SD) and differentiation (SDiff) in zebrafish, two processes that are still not fully understood (for reviews see: [11–13]). Unlike the ZZ/ZW chromosome-based process of wild zebrafish strains [14], SD of domesticated lines relies on a polygenic system with environmental factors playing secondary roles [11, 12, 14, 15].

Irrespective of their eventual sex, zebrafish larvae initially develop a “juvenile ovary” giving rise to early oocytes [16, 17]. The direction of gonad development is contingent on the survival of these early oocytes. In developing males they undergo apoptosis, as the juvenile ovary transforms into testis [18].

An additional feature of zebrafish sex is its reliance on primordial germ cells (PGCs) and, subsequently, gonadal germ cells (GCs). These stem cells are specified in the embryo and migrate into the gonadal ridge during the early development (for review see: [19]), where they differentiate into ovarian stem cells (OSCs; [20]) in females and spermatogonial stem cells (SSCs; [21]) in males, respectively.

The initially unimodal distribution of PGC numbers among individuals shifts to a bimodal one during early development in fish due to their differential amplification between the two sexes: larvae with high and low GC counts being prospective zebrafish females and males, respectively [22]. Experimental reduction of the PGC count during early development leads to male bias, while its increase promotes female bias [23–25], and PGC-ablated fish develop into sterile males [26, 27].

Interestingly, zebrafish genes primarily involved in genome maintenance have also been linked to SDiff. Knockout of genes associated with the Fanconi anemia (FA) pathway leads to complete or partial male bias, while masculinization can be observed in homozygous mutants for *rad51* [28–32]. This phenomenon could be attributed to increased germ cell apoptosis, as the all-male phenotypes could be rescued by the concurrent knockout of *tp53*, a central gene in apoptotic regulation [28, 29, 31, 33].

Here we show that in zebrafish *blm* loss-of-function affects both somatic and germ line cells, and Blm is necessary both during exponential proliferation of GCs in females and during meiosis in males. We also find that the *blm* phenotype is not rescued by the impairment of *tp53*.

## Results

### Generation of Blm loss-of-function zebrafish

The zebrafish *blm* ortholog is located on chromosome 18 (ENSDARG00000077089, GRCz11; Fig. 1A). Elevated levels of *blm* transcripts can be detected in the oocyte (Fig. 1B, C), but these maternal transcripts undergo rapid degradation during the mid-blastula transition and zygotic expression of the gene starts at later stages of gastrulation (Fig. 1B–D) [34, 35].

In order to gain a better understanding of the role of Blm in zebrafish, we created a mutant allele (*elu15*) using CRISPR/Cas9 by targeting the fourth exon. We were able to isolate a small indel that results in a premature stop codon (p.Thr101Leufs*4) and a severely truncated protein (Fig. 1E, F).

### Blm loss-of-function does not impair early somatic development and DNA damage sensitivity of zebrafish

**Fig. 2:**
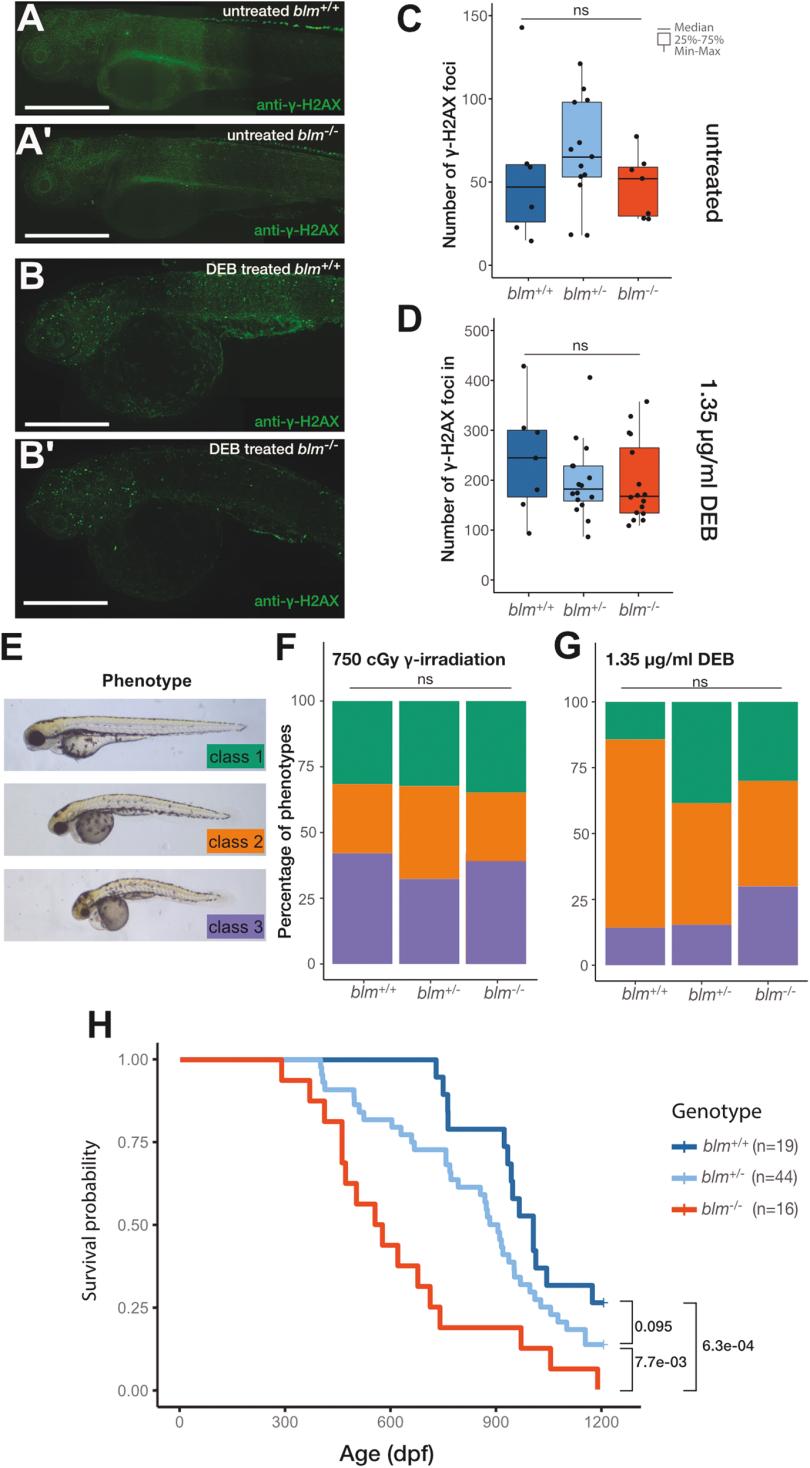
Loss of Blm function does not increase sensitivity to DNA damage, however severely reduces lifespan in zebrafish. **A**, **A**′ Anti-γ*-*H2AX labeling in the head and trunk of 3 dpf untreated wild-type (**A**) and *blm*^*−/*−^ embryos (**A**′) (Scale bar: 500 μm). **B**, **B**′ Anti-γ*-*H2AX labeling in the head and trunk of 3 dpf wild-type (**B**) and *blm*^*−/−*^ (**B**′) animals after treatment with the DNA interstrand cross-linking agent diepoxybutane (DEB) (Scale bar: 500 μm). **C**, **D** γ*-*H2AX-positive foci in the head and trunk of different *blm* genotypes under control circumstances (**C**) and after DEB treatment (**D**) (ns not significant). **E** Phenotypes observed after gamma irradiation: Class 1—wild-type; Class 2—mild necrosis in the tectum and smaller eyes; Class 3—heavy necrosis all over the body, heart edema. Untreated controls were all Class 1. **F**, **G** The distribution of phenotype severity following gamma irradiation (**F**) and DEB treatment (**G**) (ns not significant). **H** Survival probability graphs of different *blm* genotypes. (Pairwise *p* values were calculated with the log-rank test, using Benjamini & Hochberg adjustment. dpf days post-fertilization).

The overall development and viability of *blm*^−*/−*^ embryos and larvae was not compromised under normal conditions, as they were present in Mendelian ratios in *blm*^*+/−*^ incross progenies (see below). Immunostaining for the phosphorylated H2A.X histone variant γ*-*H2AX, a marker of double-stranded DNA breaks (DSBs) did not reveal differences between *blm*^*−/*−^ embryos and their siblings at 3 days post-fertilization (dpf) (Fig. 2A, B′ and Supplementary Fig. 1). This result is strikingly different from the stark increase of DSBs observed in *rad51* mutants [28].

The lack of differences in the number of DSBs prompted us to test the effects of different forms of genotoxic treatment in these animals. First, we applied 750 cGy γ-radiation to induce DSBs in the DNA and assayed larvae for phenotypic differences 1-day post-treatment. Again, we observed no significant differences in the phenotypic distribution of irradiated embryos (Fig. 2E–F). Next, we applied diepoxybutane (DEB), a reagent with well-characterized DNA–DNA and DNA–protein cross-linking effects [29]. DEB treatment resulted in an overall increase in the number of γ*-*H2AX punctae in the treated embryos (Fig. 2C, D and Supplementary Fig. 1), but no discernible differences were observed between embryos of different *blm* genotypes (Fig. 2D). Furthermore, while the treatment of embryos from 4 to 72 h post-fertilization with DEB resulted in phenotypes similar to those observed after γ-radiation, *blm*^*−/*−^ embryos showed no significant increase in the proportion of more severe phenotypes as compared to heterozygotes or wild types (Fig. 2G).

Overall, we observed no short-term somatic effects of Blm deficiency under either normal or genotoxic conditions.

### Blm mutant zebrafish show markedly shortened lifespan

In view of the fact that reduced average lifespan is one of the most striking consequences of BSyn, we set out to conduct a longevity assay on the offspring of *blm*^*+/−*^ fish. We found that while the incidence of observable malignancies did not increase, *blm*^*−/*−^ homozygous mutant fish lived markedly shorter than wild-type animals (Fig. 2H). While *blm*^*+/*−^ heterozygotes also appeared to have a somewhat reduced lifespan compared to their wild-type siblings, this effect was not significant (Fig. 2H).

### All *blm* mutant zebrafish develop into males with fertility defects

**Fig. 3:**
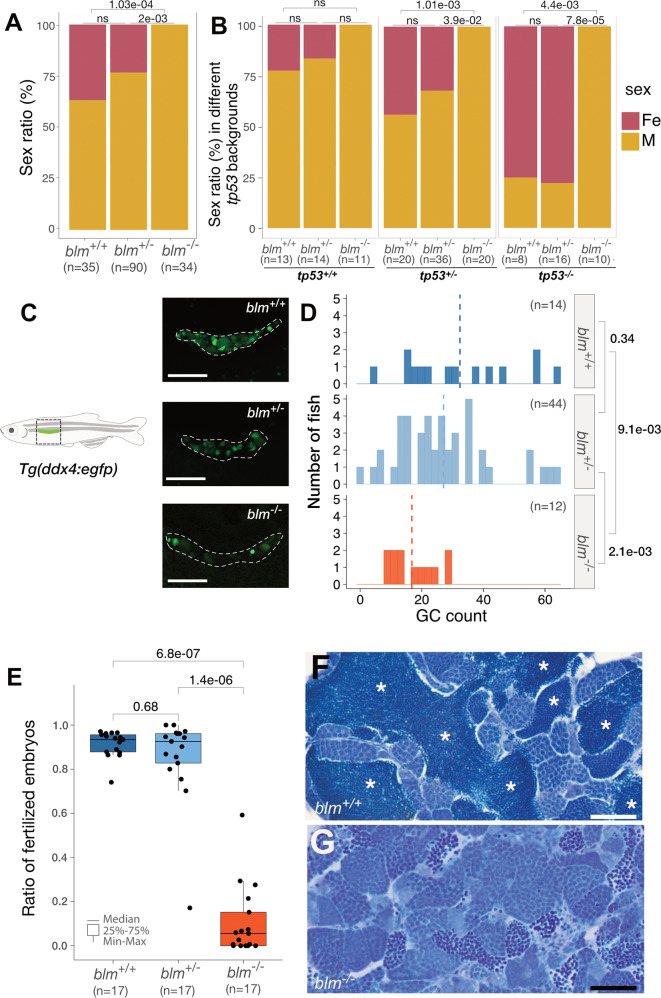
Homozygous *blm* mutants develop into males with fertility defects. **A** The sex ratio of offsprings derived from *blm*^*+/*−^ incrosses suggests a role for Blm in zebrafish sex determination and/or gonad differentiation. Data from two incrosses combined is shown. (For individual incrosses, see Supp Fig. 2A). **B** Complete lack of females among *blm*^*−/*−^ mutant fish in the absence of a functional Tp53 indicates that the effect of Blm is Tp53-independent. (Pairwise *p* values were calculated with Chi-square test; ns not significant. Data from two incrosses combined are shown. For individual incrosses, see Supplementary Fig. 2B). **C** The number of GFP-positive germ cells (GCs) in the gonad of *blm*^+/+^ and *blm*^+/−^ individuals was substantially higher than that of their *blm*^−/−^ siblings. All individuals were on a *Tg(ddx4:egfp)* background at the age of 1-month post-fertilization (scale bar: 100 μm). **D** Gonadal GC counts of different *blm* genotypes as determined on a *Tg(ddx4:egfp)* background at the size of 5–6 mm. While GC counts of heterozygous *blm*^*+/*−^ individuals were similar to those of wild types, homozygous *blm*^*−/−*^ individuals showed significantly lower numbers (*p* = 2.5e-03; Welch *t*-test). **E** The ratio of fertilized embryos in a total of 51 crosses using males of different *blm* genotypes. **F**, **G** Toluidine Blue staining of testis sections from wild-type (**F**) and *blm*^−*/*−^ mutant (**G**) fish. Asterisks denote spermatozoa clusters of normal densities. Homozygous mutant *blm*^−*/−*^ testes showed a drastic reduction of mature spermatozoa (scale bar: 50 μm). Labels: Fe female, M male, GC germ cell.

Blm impairment did not result in compromised early viability and *blm*^*−/−*^ zebrafish individuals were present at expected ratios at 3 months of age. However, similar to results reported previously for most FA proteins and Rad51, complete impairment of Blm resulted in all fish developing as males with drastically reduced fertility (Fig. 3A and Supplementary Fig. 2). In order to reveal if the phenotype is linked to a failure in the expansion of the PGC compartment (as seen for other mentioned mutants), we crossed our *blm*^*+/−*^ carriers into the *Tg(ddx4:egfp)* background, where the increased expression of GFP in germ cells (GCs) of the two sexes [36] and subsequent differential accumulation of its protein product in the two gonad types [17] allow for clear identification of the two sexes from 4–6 weeks onwards. In accordance with an all-male development, every *blm*^*−/*−^*;Tg(ddx4:egfp)* individual displayed little or no gonadal expression of the transgene at the age of one month, suggestive of testis development (Supplementary Fig. 2).

In order to discern whether this phenomenon was due to precocious apoptosis of GCs in the mutant fish, we analyzed the sex ratio in *blm*^*−/−*^*; tp53*^*M214K*^ [37] double mutants and found that they also yielded exclusively males (Fig. 3B and Supplementary Fig. 2). This suggests that the complete masculinization of these double mutants was the result of a GC-loss due either to Tp53-independent cell death or to other processes not involving cell death at all.

One possible explanation for the all-male *blm*^*−/−*^ phenotype is that due to the impaired cell proliferation GC numbers in the germ lines of mutant homozygotes fail to exceed the threshold necessary for female development [24]. To test this hypothesis, we counted gonadal GCs in 2-week old *blm*^*−/−*^*;Tg(ddx4:egfp)* fish. At this stage, lower GC numbers can be typically observed in those wild-type individuals that will eventually develop into males and higher ones in future females [23, 24]. Indeed we found that in the progeny of *blm*^*+/*−^ carriers, homozygous mutants had significantly reduced GC numbers compared to their wild-type and heterozygous siblings (*blm*^*+/+*^ (*n* = 14): 32.4 ± 18.3; *blm*^*+/−*^ (*n* = 47): 25.4 ± 16.3; *blm*^*−/−*^ (*n* = 14): 14.4 ± 9.02) (Fig. 3C, D).

Despite the similarity between all-male phenotypes of *blm* and *fanc* mutants, we found that all *blm*^*−/*−^ males were almost completely sterile (Fig. 3E), a feature that differs from that of *fanc* loss-of-function mutants [28, 29]. *Blm*^−*/*−^ fish also displayed aberrant testis morphology (Supplementary Fig. 3) and histological analysis revealed an impairment in the formation of mature spermatozoa (Fig. 3F, G).

Zebrafish undergo a cystic form of spermatogenesis, where single SSCs undergo mitotic clonal expansion and a final wave of meiosis to form mature spermatids [38, 39]. While in *blm*^*−/−*^ males cysts of different sizes comprising a differing number of spermatogonia were present, post-meiotic spermatid clusters were extremely rarely observed (Fig. 3F, G).

### Blm-deficient spermatocytes are stuck in meiotic prophase I

**Fig. 4:**
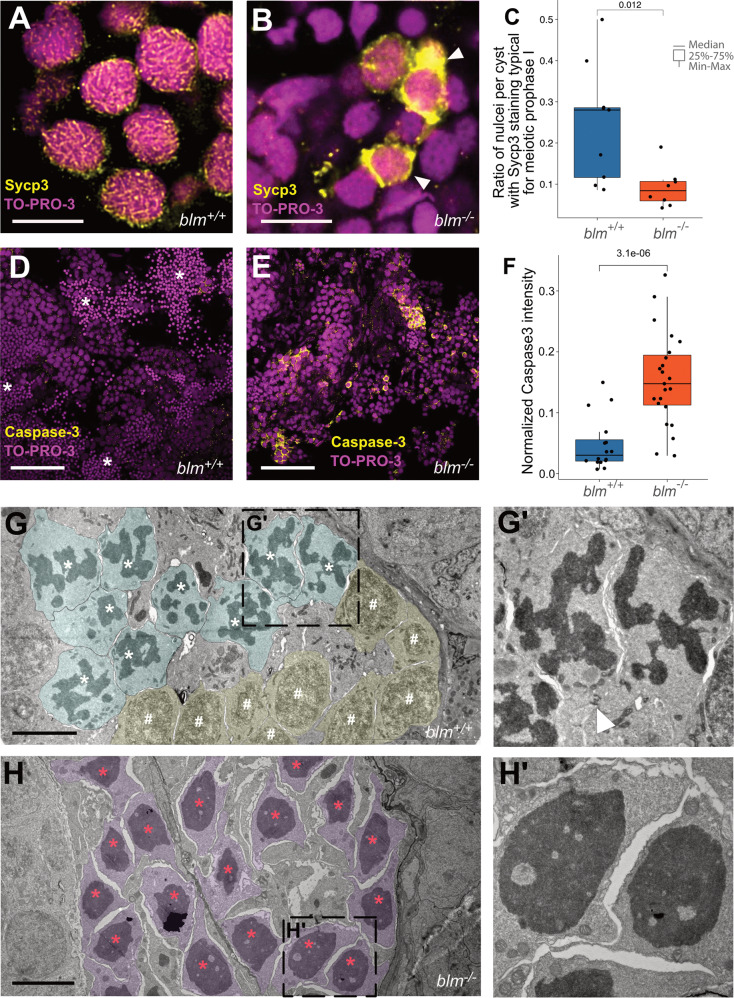
Blm loss-of-function results in meiotic defects during spermatogenesis in zebrafish males. **A**, **B** Representative Sycp3 immunostainings of wild-type and *blm*^*−/*−^ spermatogonial cysts. In wild-type cysts (**A**) patterns typical for meiotic prophase I can be detected, whereas in mutant cysts (**B**) aberrant Sycp3 patterns (white arrowheads) can be observed (*n* = 8 different wild-type and *blm*^*−/−*^ samples were tested each). TO-PRO-3 staining (red) denotes nuclei (scale bar: 8 μm). **C** Ratio of nuclei showing Sycp3 immunostaining patterns characteristic for pachytene. A comparison of wild-type and mutant testes suggests that *blm*^−*/*−^ are defective in entering pachytene. **D**, **E** Cells undergoing programmed cell death as shown by Caspase-3 (green) staining in wild-type (**D**) and *blm*^*−/−*^ testes (**E**). TO-PRO-3 staining (red) denotes nuclei (*n* = 6 different wild-type and *blm*^*−/*−^ testis lobes were tested each). Asterisks denote spermatozoa clusters (Scale bar: 50 μm). **F** Normalized Caspase-3 stainings of wild-type and mutant testes suggest increased apoptosis in the absence of Blm. **G**, **G**′ Electron microscopic images of representative spermatogenic cysts with spermatocytes in meiotic prometaphase (cyan) and other early stages of meiosis I (yellow) from wild-type males. White asterisks denote condensed chromosomal structures, white hash symbols indicate nuclei in early meiotic prophase I (see Zhang et al., 2014). The white arrowhead in **G**′ indicates a cytoplasmic bridge between spermatocytes (scale bar: 5 μm). **H**, **H**′ Electron micrographs of spermatogenic cysts of *blm*^*−/*−^ mutant males with spermatocytes (purple) showing aberrantly condensed chromatin (red asterisk), which was found to be the characteristic feature of mutant testes. Note that there are no nuclear envelopes observed around chromatin condensates, indicating that this phenomenon is related to cell division (see also Supplementary Fig. 5) (scale bar: 5 μm).

Blm has been implicated in multiple steps of the meiotic process [40]. Therefore, we sought to understand whether the drastically reduced number of spermatids in the testis of *blm*^*−/−*^ males is due to defective meiosis. Staining with anti-γ*-*H2AX suggests that unlike in *spo11* mutants [41], in *blm*^*−/*−^ testes DSB formation is not affected (Supplementary Fig. 4). We examined meiosis using an antibody specific for Sycp3, a component of the synaptonemal complex, previously shown to accumulate during the meiotic prophase [41]. While in the testes of wild-type fish spermatids showed a typical mix of Sycp3 staining patterns (e.g. pachytene, Fig. 4A), in mutants nuclei either displayed Sycp3 patterns typical for leptotene or showed an aberrant accumulation of Sycp3 in the nuclear periphery (Fig. 4A–C). The latter pattern has been observed before in apoptotic spermatids of rats [42], suggesting that in *blm*^*−/−*^ mutants many spermatogonia and spermatocytes undergo programmed cell death. Supportive of this, in our *blm*^*−/−*^ mutants spermatocytes in spermatogenic cysts often undergo apoptosis as visualized by Caspase-3 staining (Fig. 4D–F). This phenotype could be attributed to the well-documented loss-of-function of BLM in the dissolution and/or resolution of double Holliday junctions [43–46].

Finally, we performed electron microscopic (EM) imaging of germ cells. In single spermatogenic cysts of wild-type and heterozygote testes we observed spermatocytes at different phases of the meiotic cell cycle (Fig. 4G, G′), often with well-delineated, clearly separated chromosomal structures (indicative of prometaphase) [47]. In *blm*^*−/*−^ mutants an aberrant chromatin condensate appeared in most spermatocytes, with no nuclear envelope, suggesting that their chromosomes failed to segregate and remained entangled (Fig. 4H, H′), a phenotype previously also observed in *Blm* mutant *Drosophila* [8].

## Discussion

Here we describe and validate a novel zebrafish model for BSyn. Our new model recapitulates several features of the human disease [1, 3], including shortened lifespan and infertility. This new line also provides important insights into the pathomechanism of the meiotic defects associated with Blm loss-of-function and expands our understanding of the formation and differentiation of zebrafish gonads.

There are several explanations for why mutations in *BLM* could affect human lifespan. Accumulation of mutations due to an inability to repair double-stranded DNA breaks that arise as a result of environmental genotoxic effects is one plausible explanation. Indeed, mutations of multiple *fanc* genes and *rad51*, all involved in DSB repair, result in an increase of cell death in zebrafish upon exposure to genotoxic agents [28, 29]. Yet, our results show that genotoxic treatments do not result in more DNA lesions or more severe phenotypes in *blm*^*−/*−^ mutant zebrafish (Fig. 2A–F). (A similar insensitivity of Blm loss-of-function to genotoxicity was also described recently for another null allele of *blm* [48]). Compared to their wild-type and heterozygote siblings, however, homozygous *blm*^*−/*−^ zebrafish have a significantly shorter lifespan (Fig. 2G).

In bacteria, RecQ is required to reduce the number of illegitimate recombination events [49–51]. In mammals, BLM is involved in the dissolution of double Holliday junctions during HR, and the processing of late replication intermediates [46, 52, 53]. The impairment in the resolution of DNA junctions might contribute to an elevated mutation rate that results both in a shortened lifespan and an increase in the number of malignancies [54–56]. Interestingly, we did not observe an increase in the incidence of cutaneous tumors in *blm*^*−/*−^ mutants and *blm*^*+/−*^ carriers during our lifespan analysis (not shown), although occasional testicular tumors have been observed.

**Fig. 5:**
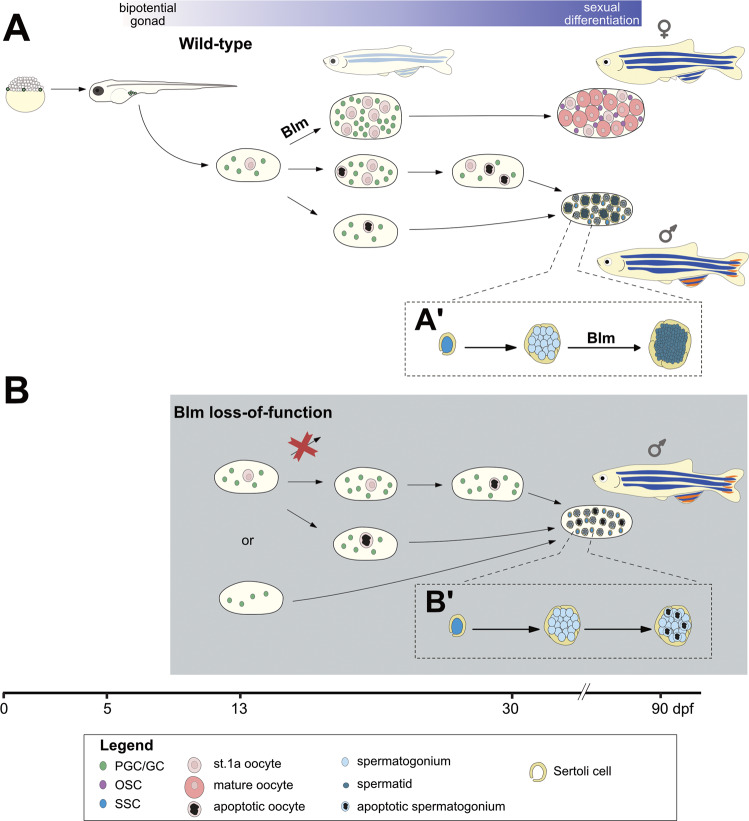
A model for the multiple roles of Blm during zebrafish gonadal development. During the early stages of ontogenesis *ddx4* (formerly *vasa*) expressing primordial germ cells (PGC) proliferate and migrate from their initial positions adjacent to the yolk syncytial layer to the gonadal mesoderm. There their proliferation continues, while a varying number of these GCs differentiate into stage 1a oocytes resulting in the development of the “juvenile ovary”. **A** In wild-type fish the number of GCs may further increase to the point where it crosses the threshold required for female fate-determining pathways to activate, resulting in the development of mature ovaries with GC-derived ovarian stem cells (OSC) and mature oocytes. If the threshold is not reached either due to insufficient GC proliferation, or none at all, a major shift in the gonadal environment occurs through which oocytes (and possibly even a subset of GCs) are eliminated via apoptosis. Subsequently, the formation of spermatogonial stem cells (SSC) and Sertoli cells occurs either by (i) differentiation of common OSC and SSC precursors (gonadal stem cells) into SSCs that in turn induce Sertoli cell differentiation in the soma; or (ii) by the transdifferentiation of somatic follicular cells into Sertoli cells that promote SSC differentiation and expansion from gonadal stem cells, thus leading to testis development. **A’** In the wild-type testis, SSCs are enveloped by Sertoli cells where they further differentiate into spermatogonia. In this structure, known as a cyst, spermatogonia go through mitotic expansion followed by synchronous meiosis into spermatids. **B** In Blm loss-of-function individuals, the complete lack of females might be caused by two major factors: (i) the number of GCs never reaches the threshold necessary for female development; and/or (ii) meiotic processes necessary for the formation of oocytes might be compromised in absence of Blm. As we do not know whether the gonads of *blm*^*−/−*^ zebrafish do make the initial “detour” through the juvenile ovary stage or not, both possibilities are indicated. **B**′ A potential explanation for the subfertility of *blm*^*−/−*^ males is that in the absence of Blm accurate DNA repair during spermatogonial meiosis is hindered, and therefore cell death is induced.

We were also able to demonstrate multiple roles for Blm in zebrafish gametogenesis and SD/SDiff (Fig. 5), as *blm*^*−/−*^ individuals invariably develop into males with severely compromised fertility (Fig. 3A, E). The same phenotype was also observed in the recently described *blm*^*cu53*^ allele as well [48]. Homozygous fish that carry this 5-bp insertional allele in the second exon of their *blm* gene also develop into almost completely sterile males, with the number of haploid spermatozoa apparent in histological sections greatly reduced.

In zebrafish one of the most important elements of SD/SDiff is the number of PGCs, GCs, and the survival of early-stage oocytes that start to form around 10 dpf in the developing gonads. If the GC population fails to undergo an exponential (type II) proliferation [22], or the early-stage oocytes die through apoptosis during metamorphosis, the “juvenile ovary” almost invariably transforms into a testis [23, 24, 31]. Replication stress that naturally arises in some dividing GCs during mitosis normally would activate DNA repair pathways, and the impairment of these pathways leads to a failure in GC expansion [57] resulting in a female-to-male sex reversal phenotype in zebrafish (Fig. 5) [28–30, 32]. Additional experiments will be necessary to find out whether or not the gonads of *blm*^*−/−*^ zebrafish do make the initial “detour” through the juvenile ovary typical for normal development.

One important difference, however, between the sex reversal observed in *blm*^−*/−*^ zebrafish compared to that of *fancd1/brca2*, *fancj*, and *rad51* mutants [28–30, 32] is that the all-male phenotype for all the latter is dependent on Tp53 function. In contrast, all *blm*^*−/−*^*;tp53*^−*/−*^ double mutants developed as males (Fig. 3B). This is reminiscent of the phenotype observed upon the impairment of the Ddx4/Vasa RNA helicase, where besides the *tp53*-independent male bias, a failure of spermatocytes to progress beyond pachytene was also described [58]. We hypothesize that in the absence of Blm DNA damage can accumulate in some fast proliferating GCs leading to mitotic arrest and spindle checkpoint activation. For unknown reasons, however, Blm loss-of-function does not appear to trigger Tp53 activation and it might lead to apoptosis probably through the activation of one of the Tp53-homologs, for example, TAp73 [59]. Alternatively, the early formation of stage 1a oocytes in the developing gonad might be impaired due to the involvement of Blm in the meiotic process, and in the absence of these cells, the gonad might not be able to develop into an ovary [31].

The role of Blm in meiosis is well established, and fertility problems have been also reported for BSyn patients of both sexes [1, 3]. In *C. elegans* BLM (HIM-6) is required to convert licensed crossover (CO) designated sites into bona fide COs and in *him-6* mutants abnormal chromosomal segregation can be observed [43–45]. Similarly, loss of *DmBlm* results in an excessive number of COs, nondisjunction, and aneuploidy during *Drosophila* meiosis as well [8]. In mice, *Blm* expression is upregulated during the leptotene stage [60] and its impairment leads to defects in meiotic progression due to the improper pairing and synapsis of homologous chromosomes and BLM-deficient cells often undergo apoptosis during spermatogenesis [61]. This latter phenotype is highly reminiscent of what we observe in the testes of our *blm*^*−/−*^ mutant zebrafish (Fig. 4).

Similar to Blm, some factors are not only involved in mitotic DNA repair but also have important roles in regulating HR during meiosis [62]. The impairment of these proteins, such as Fancd1/Brca2, Fancj, and Rad51 leads to defects in both cell division types. Thus, similarly to *blm*^*−/−*^ mutants, both sex reversal and male subfertility can be observed in the mutants of the genes that encode these factors [28–30, 32]. In contrast, only subfertility was observed in the mutants of *hsf5, mlh1*, and *spo11*, all of which are involved in the meiotic process (Fig. 5A) [41, 63, 64].

The viability and relatively mild somatic phenotype of Blm loss-of-function suggests that there is redundancy with other RecQ orthologs during the division of somatic cell progenitors. Based on their expression profiles (Fig. 1B) *recql* and *recql5* could have important roles during embryogenesis, but it remains to be determined how embryonic and larval development (and viability) are affected when these genes are mutated. Also, while the expression of *blm* is clearly elevated in zebrafish oocytes, *recql4* has a similar expression pattern (Fig. 1B) [34, 35]. It also remains to be seen to what extent are their functions redundant during gametogenesis.

## Materials and methods

### Zebrafish husbandry and welfare

Wild-type (AB), and *blm*^*elu15*^ and *tp53*^*M214K*^ [37] mutant zebrafish in non-transgenic and *Tg(ddx4:egfp)* [36] backgrounds used in this study were maintained in the fish facility of ELTE Eötvös Loránd University according to standard protocols [65, 66]. All experimental procedures were approved by the Hungarian National Food Chain Safety Office (Permit Numbers: PE/EA/290-2/2018 and PE/EA/2025-7/2017). Fish involved in the lifespan assay were reared at similar stocking densities.

A detailed description of CRISPR-based mutagenesis, fertility assays, and genotoxic treatments can be found in Supplementary Methods.

### Histology

For histological examination, fish were dissected and their testes were fixed by a fixative containing 4% paraformaldehyde. The samples were embedded using JB-4 resin (Merck-Sigma, EM0100) as described in the manufacturer’s protocol. About 2–5-μm-thin sections were made by rotation microtome and stained with Toluidine Blue. Sections were photographed using a Zeiss Axio Imager microscope system.

For immunostaining we used the anti-Sycp3-rabbit (Abcam, ab15093; 1:500 dilution), anti-Caspase-3-rabbit (Cell Signaling Technology, 9661; 1:500 dilution) and anti-γ-H2AX-rabbit (GeneTex, GTX127342; 1:1000 dilution for larvae, 1:200 dilution for testes) primary antibodies in combination with anti-rabbit-Alexa488 (Invitrogen, A-11008; 1:200 dilution) secondary antibody. Nuclei were stained with TO-PRO-3 (Invitrogen, T3605; 1:3000 dilution). Larvae stained with anti-γ-H2AX were imaged under a Zeiss Axio Imager M2 using an EC Plan-NeoFluar 10 × 0.3 objective, and a Colibri 7 LED light source.

For electron micrography we fixed the samples using 3.2% PFA, 0.2% glutaraldehyde, 1% sucrose, 40 mM CaCl_2_ in 0.1 M cacodylate buffer. Post-fixation was performed with 1% ferrocyanide-reduced osmium [67] and samples were embedded into an epoxy resin medium. Ultrathin sections were counterstained with 2% uranyl acetate solution and Reynolds’s lead citrate solution. For examination, a JEOL JEM 1011 transmission electron microscope was used, equipped with a Morada 11-megapixel camera.

A detailed description of in situ hybridization, PGC quantification, and image processing can be found in Supplementary Methods.

## Supplementary information

Supplemental Information

CDD Checklist file

Email confirmations for the change in the author list compared to the original submission.

## Data Availability

All data needed to evaluate the conclusions are present in the main article and the supplementary information. Additional datasets related to this paper may be requested from the corresponding authors.
