# Supplementary material for: Bloom syndrome helicase contributes to germ line development and longevity in zebrafish
Source: Cell Death Dis. 2022 Apr 18;13(4):363. doi: 10.1038/s41419-022-04815-8 (PMC9016072; doi:10.1038/s41419-022-04815-8)
Supplement: Supplementary file 1 — Supplemental Information [file 41419_2022_4815_MOESM1_ESM.docx]

**SUPPLEMENTARY INFORMATION**


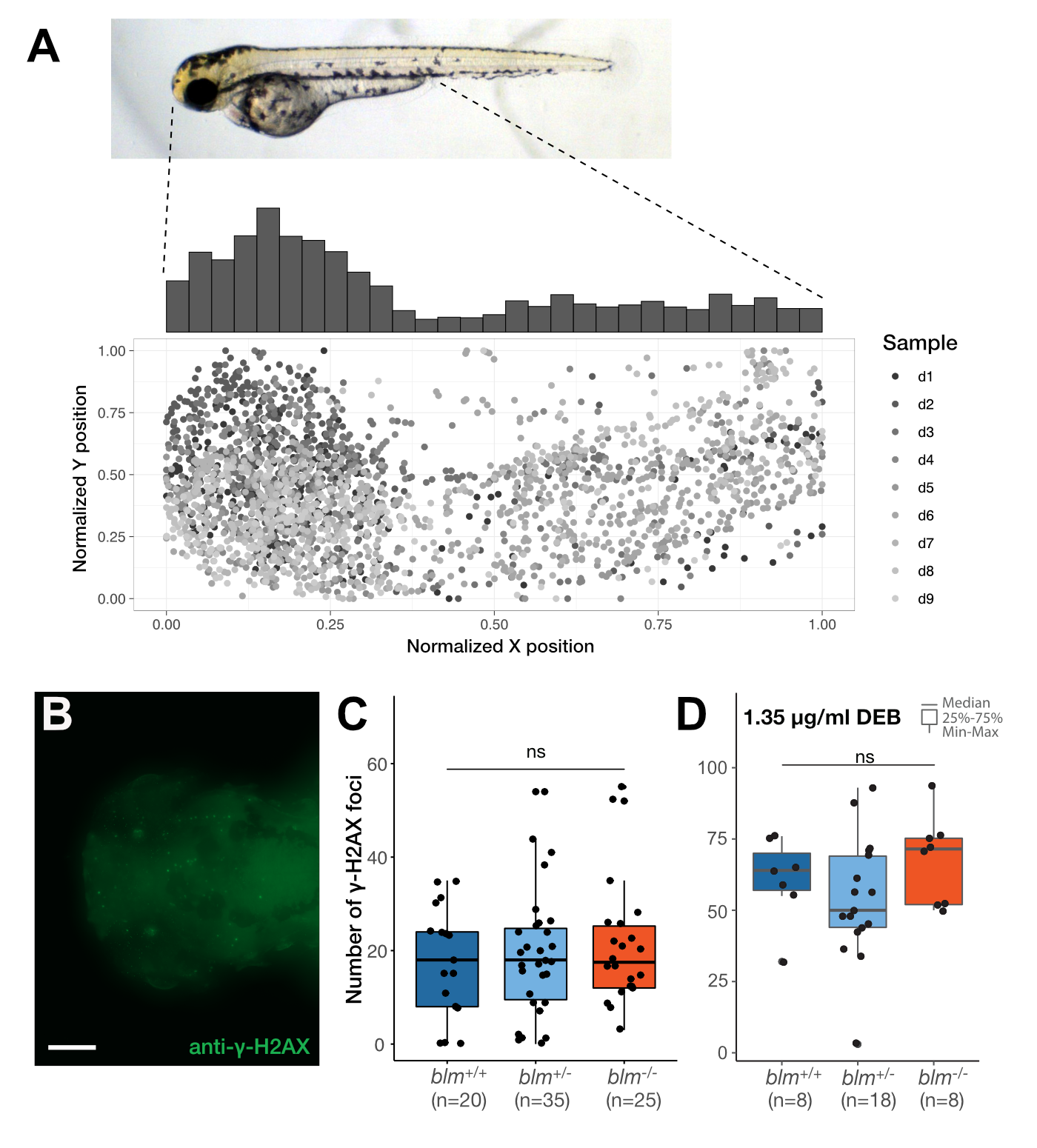


**Supplementary Figure 1: DEB induces the accumulation of DSBs especially in the head region, but lack of Blm does not affect the efficiency of DSB-repair.** (A) The distribution of γ*-*H2AX-positive foci along the body of zebrafish embryos treated with DNA interstrand cross-linking agent diepoxybutane (DEB) shows a clear skew towards the head region. (Untreated zebrafish embryo shown for illustration.) (B) Anti-γ*-*H2AX labelling in the head of a wild type embryo. (Dorsal view, 2 dpf, anterior to the left; scale bar: 100 μm). (C,D) γ*-*H2AX-positive foci in the head of different *blm* genotypes under control circumstances (C) and after treatment with DEB (D). (ns – not significant)

**
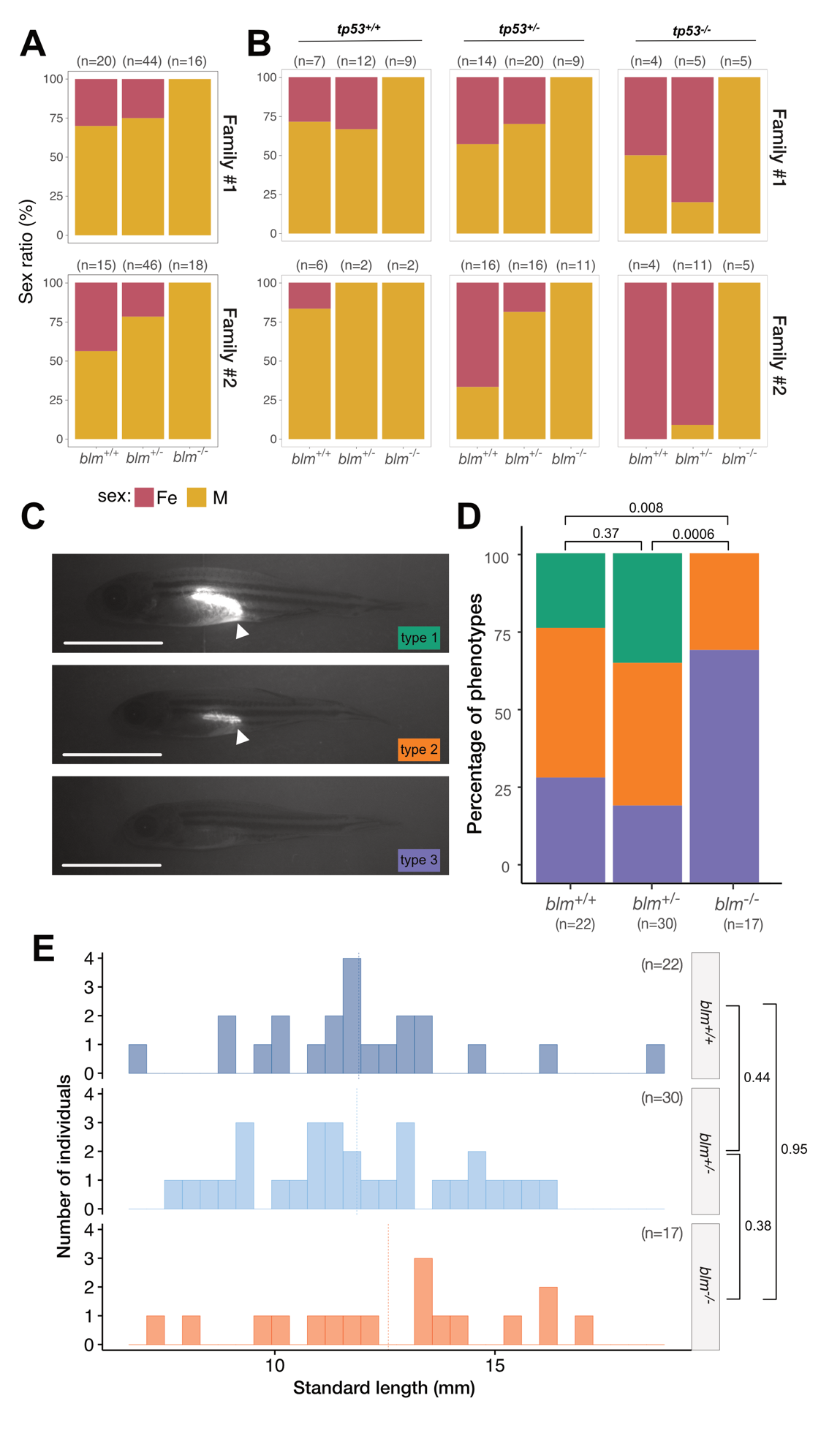
**

**Supplementary Figure 2: SD and GC proliferation is compromised in the absence of Blm.** (A) In two independent families arising from a *blm^+/-^* incrosses all *blm^-/-^* fish developed as males. (B) All male development of *blm^-/-^* fish was also observed regardless of the *tp53* background in two independent families. Importantly, in both families, *tp53* loss-of-function resulted in a pronounced feminization of *blm^+/-^* and *blm^+/+^* fish, but complete Blm impairment was epistatic to this effect. (C) At one month of age the gonads of *Tg(ddx4:egfp)* zebrafish show either strong fluorescence (type 1), weak fluorescence (type 2) or no fluorescence (type 3). (Scale bar: 5 mm). (D) While in *blm^+/+^;Tg(ddx4:egfp)* and *blm^+/-^;Tg(ddx4:egfp)* zebrafish similar number of individuals exhibit strong GFP signal in their gonads to those without gonadal GFP, gonads of *blm^-/-^;Tg(ddx4:egfp)* fish very seldom show fluorescence. (Pairwise *p*-values were calculated using Welch *t*-test.) (E) The growth of *blm^-/-^* fish is not impaired compared to their siblings, as measured by standard length at 1 month age.


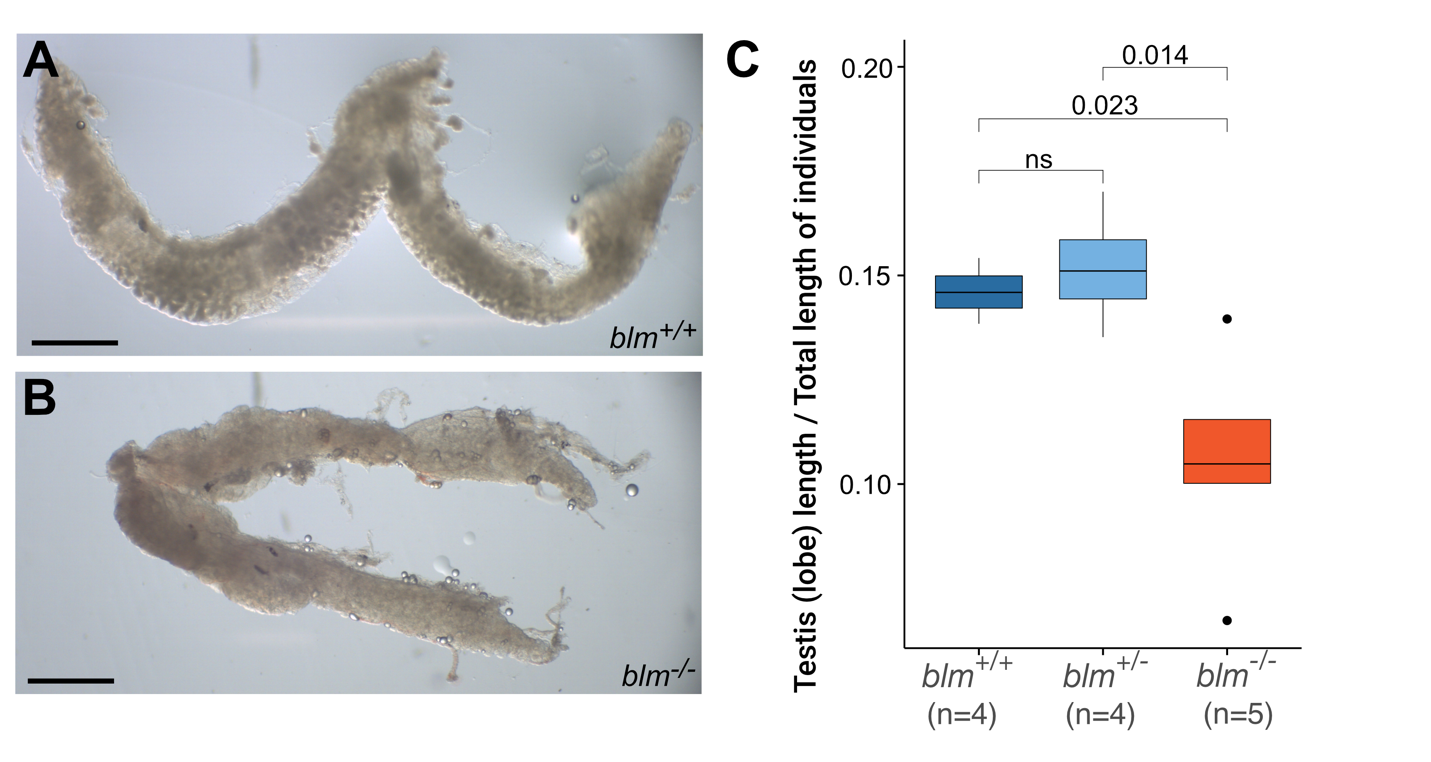


**Supplementary Figure 3: Testes of *blm* mutants are smaller and show abnormal morphology.** Compared to wild-type testes (A) those of *blm^-/-^* individuals (B) showed less pronounced seminiferous tubule structure under transmitted light stereo microscopy and were significantly shorter (C). (Pairwise *p*-values were calculated using Welch *t*-test. Scale bar: 1 mm)

**
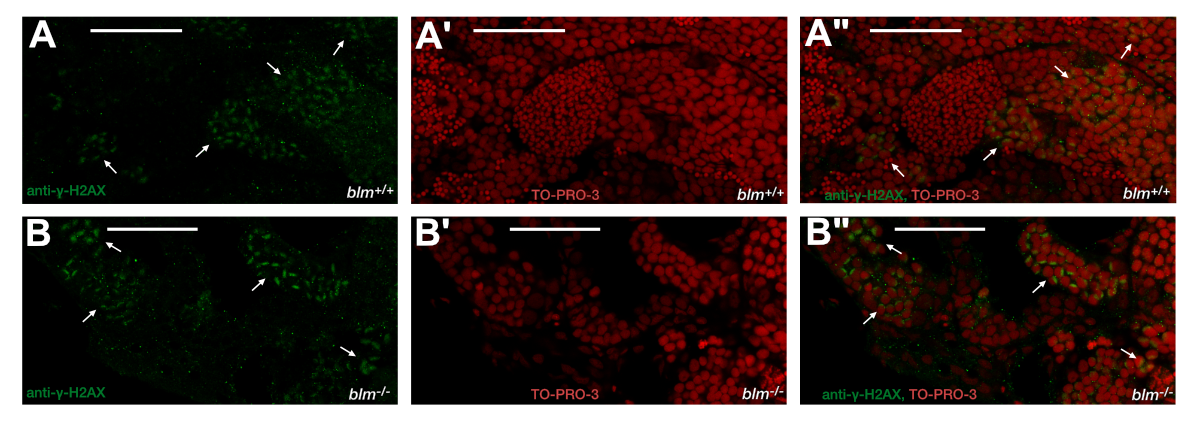
**

**Supplementary Figure 4: The formation of double-stranded breaks (DSBs) can be observed in meiotic spermatogonia of *blm^-/-^* mutants**. (A-A”) Wild-type testes. (B-B”) Testes of *blm^-/-^* mutants. Labels: Anti-γ-H2AX (green); TO-PRO-3 (nuclear; red). White arrows show clusters of spermatogonia entering meiotic prophase I with DSBs. (Scale bar: 50 μm)


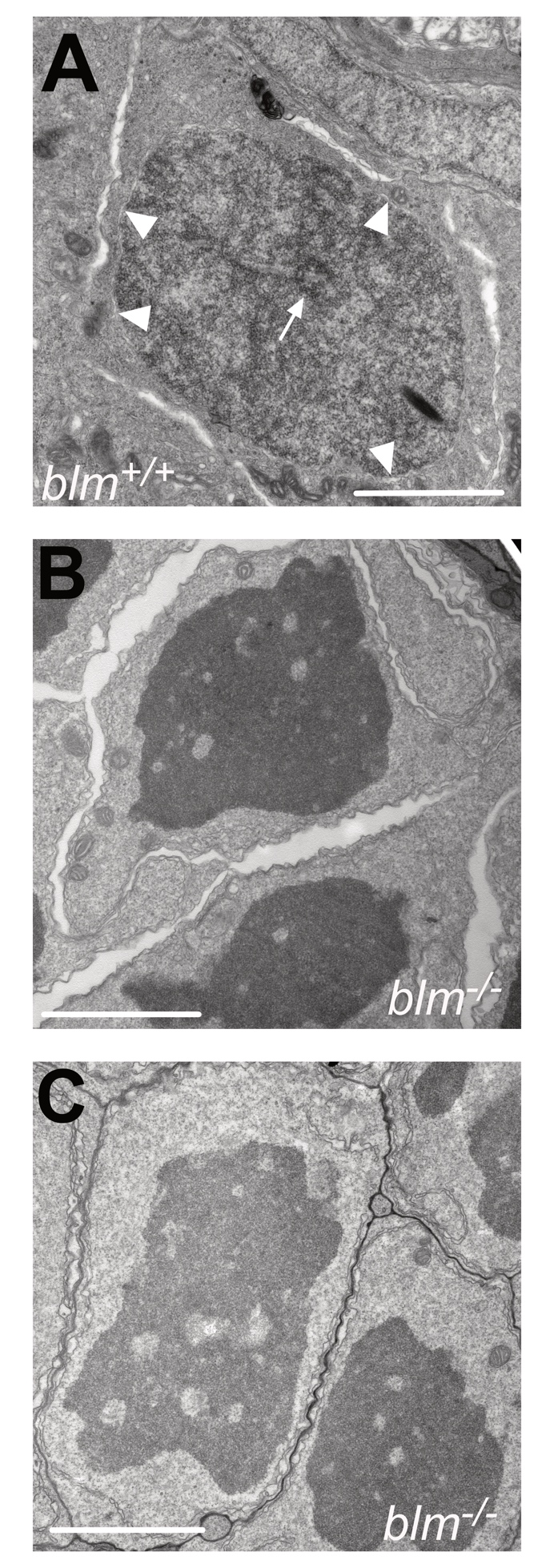
**Supplementary Figure 5: EM images of representative spermatocytes from wild type and *blm^-/-^* mutant animals.** (A) A typical wild type spermatocyte in early meiosis I. Note the presence of the nuclear envelope (white arrowheads) and a structure resembling a synaptonemal complex (white arrow). (B and C) Typical spermatocytes from mutants show large chromosomal condensates, with no visible nuclear envelope. (Scale bar: 2 μm).

**Supplementary Methods**

*CRISPR/Cas9 mutagenesis and genotyping*

SgRNA targeting the sequence 5’-AAGACAAATACAGTTAATCC-3’ in the 4^th^ exon of *blm* was synthesized and injected as described before [1]. In the P0 generation we isolated mosaic fish that carried in their germline the c.302_304delACAAinsTT indel mutation. These carriers were outbred with AB wild-type individuals. Genomic DNA isolation for genotyping of fin clips from adults was carried out as described before [2]. Genotyping for the *blm^elu10^* allele was accomplished via allele-specific PCR. Two reactions were assembled per sample, one with wild-type specific (5’-CGCCCAGCAAACCGAAGACAA-3’) and another with mutant specific (5’-GCGCCCAGCAAACCGAAGTTA-3’) forward primers. In both cases the reverse primer was: 5’-GTATCCGGTGAAACACAGTGGA-3’. Genotypes were determined from gel electrophoretic analysis. Genotyping of the *tp53^M214K^* allele was performed by Sanger sequencing the PCR product created with the following forward and reverse primers: 5'-TTGCCAGAGTATGTGTCTGTCC-3' and 5'-CAGCATCATGAAGCATCAAA-3', respectively.

*Genotoxic treatments*

γ-irradiation of fish was carried out as described before [3] at the Basic Medical Science Center of Semmelweis University, Budapest. DEB treatments were performed as described before [4]. Embryos were allocated randomly to the treatment and control groups. Phenotypic scoring of individual embryos was carried out prior genotyping in a blinded manner by another member of the team.

*Fertility assay*

Male fish for all *blm* genotypes were individually bred with wild-type *AB* females of similar age (8 months post-fertilization). Fish were paired at random in repeated attempts with at least 5 days of rest between breeding sessions. Collected eggs were analyzed at least 6 hours post-fertilization, based on the occurrence of epiboly. Decomposing eggs were excluded from statistical analysis, as success of fertilization could not be determined.

In situ *hybridization*

*In situ* hybridizations were performed as described before [5]. A ~1 kb fragment of the *blm* cDNA was cloned using primers blm-F – 5’- GGAGTCGAAACACCTGGTGGTA-3’ and blm-R – 5’- CTCATCAATGACCAAGCGAGCC-3’ into pGEM-T Easy vector (Promega) following the manufacturer’s protocol, cut with SacII and transcribed using SP6 polymerase.

*GFP-positive GC quantification*

The offspring of *blm^+/-^;Tg(ddx4:egfp)* fish were raised to 5-6 mm standard length and were euthanized and fixed in 4 % PFA in PBS. Larvae were screened for GFP expression under a Zeiss SteREO Lumar.V12 microscope equipped with a NeoLumar S 0.8x objective and a CoolLED pE-300lite light source. GFP-positive specimens were embedded in 2 % low melting point agarose gel and were imaged under a Zeiss Axio Imager M2 upright microscope with semi-confocal ApoTome 2.0 using an EC Plan-NeoFluar 10x 0.3 objective and a Colibri 7 LED light source. Optical sections of fish were imaged unilaterally at 1 μm intervals. The number of slices imaged was determined on a case-by-case basis and was dependant upon the size of the given larva and the position of observable GFP-positive cells within it (41-178 stacks). GFP-positive germ cells were enumerated manually in Zeiss ZEN, whereby a single operator determined their number blindly with regards to genotype. All enumeration procedures were carried out by the same operator for the purpose of consistency. Wherever possible, care was taken not to count clumps of cells as a single cell. Cell size and intensity of fluorescence were not taken into consideration.

*Statistics and visualization*

Multiple sequence alignments were performed using the Uniprot UGENE toolkit [6]. For the alignments we used sequences for the helicase ATPase and helicase C-terminal domains of the respective proteins. Statistical analysis and visualization was performed in R (R Core Team, 2018) using the *ggplot2* package [7]. To display phylogenetic relationships we used the *jsPhyloSVG* script [8]. For lifespan analysis we used the *survminer* package (v. 0.4.6, [9]).

In the case of the anti-γ-H2AX and anti-Caspase3 immunostainings, statistical analysis has been performed based on approximately 20 non-overlapping pictures with the same size. Using ImageJ software, we measured the integrated pixel density of every record for the 488 nm channel and normalized these values by the integrated pixel density value of the TO-PRO (661 nm) channel.

Image processing in ImageJ was performed as described before [10]. All figures have been assembled in Affinity Designer (Serif Europe).

**Supplementary References**

1. Gagnon JA, Riley B, Valen E, Thyme SB, Huang P, Ahkmetova L, et al. Efficient mutagenesis by Cas9 protein-mediated oligonucleotide insertion and large-scale assessment of single-guide RNAs. PLoS ONE. 2014;9(5):e98186.

2. Meeker ND, Hutchinson SA, Ho L, Trede NS. Method for isolation of PCR-ready genomic DNA from zebrafish tissues. BioTechniques. 2007;43(5):610-612–4.

3. Botthof JG, Bielczyk-Maczyńska E, Ferreira L, Cvejic A. Loss of the homologous recombination gene rad51 leads to Fanconi anemia-like symptoms in zebrafish. Proc National Acad Sci USA. 2017;114(22):E4452–61.

4. Ramanagoudr-Bhojappa R, Barsh GS, Carrington B, Ramaswami M, Bishop K, Robbins GM, et al. Multiplexed CRISPR/Cas9-mediated knockout of 19 Fanconi anemia pathway genes in zebrafish revealed their roles in growth, sexual development and fertility. PLoS Gen. 2018;14(12):e1007821.

5. Bellipanni G, Varga M, Maegawa S, Imai Y, Kelly C, Myers AP, et al. Essential and opposing roles of zebrafish beta-catenins in the formation of dorsal axial structures and neurectoderm. Development. 2006;133(7):1299–309.

6. Okonechnikov K, Golosova O, Fursov M, team U. Unipro UGENE: a unified bioinformatics toolkit. Bioinformatics. 2012;28(8):1166–7.

7. Wickham H. ggplot2: elegant graphics for data analysis. 2016. Springer-Verlag New York. ISBN 978-3-319-24277-4, Available from: https://ggplot2.tidyverse.org.

8. Smits SA, Poon AFY, Ouverney CC. *jsPhyloSVG*: a javascript library for visualizing interactive and vector-based phylogenetic trees on the web. PLoS ONE. 2010;5(8):e12267.

9. Kassambara A, Kosinski M, Biecek P. *survminer*: Drawing Survival Curves using “ggplot2”. R package version 0.4.6. [Internet]. 2019. Available from: https://cran.r-project.org/package=survminer

10. Preibisch S, Saalfeld S, Tomancak P. Globally optimal stitching of tiled 3D microscopic image acquisitions. Bioinformatics. 2009;25(11):1463–5.
