# Supplementary material for: Bloom syndrome helicase contributes to germ line development and longevity in zebrafish
Source: Cell Death Dis. 2022 Apr 18;13(4):363. doi: 10.1038/s41419-022-04815-8 (PMC9016072; doi:10.1038/s41419-022-04815-8)
Supplement: Supplementary file 3 — Email confirmations for the change in the author list compared to the original submission. [file 41419_2022_4815_MOESM3_ESM.pdf]

From: **Máté Varga** mvarga@ttk.elte.hu
Subject: Your confirmation needed for author change in our CDDIS-21-1343 ms
Date: 23 March 2022 at 07:55
To: Mihály Dr. Kovács mihaly.kovacs@ttk.elte.hu, Dr. Orbán László Orban.Laszlo@uni-mate.hu, Jezsó Bálint jezso.balint@ttk.elte.hu, annus Tamás tamas.annus@ttk.elte.hu, gabor.harami@ttk.elte.hu
Cc: György Ullaga ullagagyuri@gmail.com, mulda@caesar.elte.hu, Dalma Meixner meixnerdalma@gmail.com

MV

Dear all,

as I am sure you are all aware, after the first revision of our Blm manuscript we have included Barnabás Németh, a MSc student who did the bulk of the genotyping experiments on the authors' list. The CDDis Editorial Office however needs your written consent to this change.

So please reply ASAP to this email, using your email address that is also used in the CDDis manucript tracking system and confirm if you agree with this change.

Best regards,
Máté

ELTE Eötvös Loránd University
Department of Genetics
Pázmány Péter stny. 1/C
H-1117, Budapest, Hungary

http://danio.elte.hu

From: Annus Tamás tamas.annus@ttk.elte.hu
Subject: Re: Your confirmation needed for author change in our CDDIS-21-1343 ms
Date: 23 March 2022 at 08:05
To: Varga Máté mvarga@ttk.elte.hu

AT

Dear Máté,

I consent to the inclusion of Barnabás Németh in the list of authors.

All the best,
Tamás

From: Varga Máté <mvarga@ttk.elte.hu>
Sent: Wednesday, March 23, 2022 12:55:51 PM
To: Dr. Kovács Mihály <mihaly.kovacs@ttk.elte.hu>; "Dr. Orbán László" <Orban.Laszlo@uni-mate.hu>; Jezsó Bálint <jezso.balint@ttk.elte.hu>; Annus Tamás <tamas.annus@ttk.elte.hu>; Dr. Harami Gábor <gabor.harami@ttk.elte.hu>
Cc: György Ullaga <ullagagyuri@gmail.com>; Müller Dalma <mulda@caesar.elte.hu>; Dalma Meixner <meixnerdalma@gmail.com>
Subject: Your confirmation needed for author change in our CDDIS-21-1343 ms

Dear all,

as I am sure you are all aware, after the first revision of our Blm manuscript we have included Barnabás Németh, a MSc student who did the bulk of the genotyping experiments on the authors' list. The CDDis Editorial Office however needs your written consent to this change.

So please reply ASAP to this email, using your email address that is also used in the CDDis manucript tracking system and confirm if you agree with this change.

Best regards,
Máté

ELTE Eötvös Loránd University
Department of Genetics
Pázmány Péter stny. 1/C
H-1117, Budapest, Hungary

http://danio.elte.hu

From: **dalma muller** dalma.muller2@gmail.com
Subject: Re: Your confirmation needed for author change in our CDDIS-21-1343 ms
Date: 23 March 2022 at 09:20
To: Jezsó Bálint jezso.balint@ttk.elte.hu
Cc: György Ullaga ullagagyuri@gmail.com, Varga Máté mvarga@ttk.elte.hu, Dr. Kovács Mihály mihaly.kovacs@ttk.elte.hu, Dr. Orbán László Orban.Laszlo@uni-mate.hu, Annus Tamás tamas.annus@ttk.elte.hu, Dr. Harami Gábor gabor.harami@ttk.elte.hu, Müller Dalma mulda@caesar.elte.hu

DM

Dear all,

I confirm that I agree with this change

Best wishes,
Dalma Müller

Jezsó Bálint <jezso.balint@ttk.elte.hu> ezt írta (időpont: 2022. márc. 23., Sze, 14:04):

> I agree with it.
>
> Regards,
> Bálint
>
> ---
>
> **Feladó:** György Ullaga <ullagagyuri@gmail.com>
> **Elküldve:** 2022. március 23., szerda 13:05
> **Címzett:** Varga Máté <mvarga@ttk.elte.hu>
> **Másolatot kap:** Dr. Kovács Mihály <mihaly.kovacs@ttk.elte.hu>; Dr. Orbán László <Orban.Laszlo@uni-mate.hu>; Jezsó Bálint <jezso.balint@ttk.elte.hu>; Annus Tamás <tamas.annus@ttk.elte.hu>; Dr. Harami Gábor <gabor.harami@ttk.elte.hu>; Müller Dalma <mulda@caesar.elte.hu>; dalma muller <dalma.muller2@gmail.com>
> **Tárgy:** Re: Your confirmation needed for author change in our CDDIS-21-1343 ms
>
> Dear all,
>
> I confirm that I agree with this change
>
> Best wishes,
> György Ullaga
>
> Máté Varga <mvarga@ttk.elte.hu> ezt írta (időpont: 2022. márc. 23., Sze, 12:55):
>
>> Dear all,
>>
>> as I am sure you are all aware, after the first revision of our Blm manuscript we have included Barnabás Németh, a MSc student who did the bulk of the genotyping experiments on the authors' list. The CDDis Editorial Office however needs your written consent to this change.
>>
>> So please reply ASAP to this email, using your email address that is also used in the CDDis manucript tracking system and confirm if you agree with this change.
>>
>> Best regards,
>> Máté
>>
>> --
>> ELTE Eötvös Loránd University
>> Department of Genetics
>> Pázmány Péter stny. 1/C
>> H-1117, Budapest, Hungary
>>
>> http://danio.elte.hu

From: György Ullaga ullagagyuri@gmail.com
Subject: Re: Your confirmation needed for author change in our CDDIS-21-1343 ms
Date: 23 March 2022 at 08:05
To: Máté Varga mvarga@ttk.elte.hu
Cc: Mihály Dr. Kovács mihaly.kovacs@ttk.elte.hu, Dr. Orbán László Orban.Laszlo@uni-mate.hu, Jezsó Bálint jezso.balint@ttk.elte.hu, annus Tamás tamas.annus@ttk.elte.hu, gabor.harami@ttk.elte.hu, mulda@caesar.elte.hu, dalma muller dalma.muller2@gmail.com

GU

Dear all,

I confirm that I agree with this change

Best wishes,
György Ullaga

Máté Varga <mvarga@ttk.elte.hu> ezt írta (időpont: 2022. márc. 23., Sze, 12:55):

> Dear all,
>
> as I am sure you are all aware, after the first revision of our Blm manuscript we have included Barnabás Németh, a MSc student who did the bulk of the genotyping experiments on the authors' list. The CDDis Editorial Office however needs your written consent to this change.
>
> So please reply ASAP to this email, using your email address that is also used in the CDDis manucript tracking system and confirm if you agree with this change.
>
> Best regards,
> Máté
>
> --
>
> ELTE Eötvös Loránd University
> Department of Genetics
> Pázmány Péter stny. 1/C
> H-1117, Budapest, Hungary
>
> http://danio.elte.hu

From: **Jezsó Bálint** jezso.balint@ttk.elte.hu
Subject: Re: Your confirmation needed for author change in our CDDIS-21-1343 ms
Date: 23 March 2022 at 09:04
To: György Ullaga ullagagyuri@gmail.com, Varga Máté mvarga@ttk.elte.hu
Cc: Dr. Kovács Mihály mihaly.kovacs@ttk.elte.hu, Dr. Orbán László Orban.Laszlo@uni-mate.hu, Annus Tamás tamas.annus@ttk.elte.hu, Dr. Harami Gábor gabor.harami@ttk.elte.hu, Müller Dalma mulda@caesar.elte.hu, dalma muller dalma.muller2@gmail.com

JB

I agree with it.

Regards,
Bálint

Feladó: György Ullaga <ullagagyuri@gmail.com>
Elküldve: 2022. március 23., szerda 13:05
Címzett: Varga Máté <mvarga@ttk.elte.hu>
Másolatot kap: Dr. Kovács Mihály <mihaly.kovacs@ttk.elte.hu>; Dr. Orbán László <Orban.Laszlo@uni-mate.hu>; Jezsó Bálint <jezso.balint@ttk.elte.hu>; Annus Tamás <tamas.annus@ttk.elte.hu>; Dr. Harami Gábor <gabor.harami@ttk.elte.hu>; Müller Dalma <mulda@caesar.elte.hu>; dalma muller <dalma.muller2@gmail.com>
Tárgy: Re: Your confirmation needed for author change in our CDDIS-21-1343 ms

Dear all,

I confirm that I agree with this change

Best wishes,
György Ullaga

Máté Varga <mvarga@ttk.elte.hu> ezt írta (időpont: 2022. márc. 23., Sze, 12:55):

> Dear all,
>
> as I am sure you are all aware, after the first revision of our Blm manuscript we have included Barnabás Németh, a MSc student who did the bulk of the genotyping experiments on the authors' list. The CDDis Editorial Office however needs your written consent to this change.
>
> So please reply ASAP to this email, using your email address that is also used in the CDDis manucript tracking system and confirm if you agree with this change.
>
> Best regards,
> Máté
> --
> ELTE Eötvös Loránd University
> Department of Genetics
> Pázmány Péter stny. 1/C
> H-1117, Budapest, Hungary
>
> http://danio.elte.hu

From: **Dr. Harami Gábor** gabor.harami@ttk.elte.hu
Subject: Re: Your confirmation needed for author change in our CDDIS-21-1343 ms
Date: 23 March 2022 at 10:23
To: dalma muller dalma.muller2@gmail.com, Jezsó Bálint jezso.balint@ttk.elte.hu
Cc: György Ullaga ullagagyuri@gmail.com, Varga Máté mvarga@ttk.elte.hu, Dr. Kovács Mihály mihaly.kovacs@ttk.elte.hu, Dr. Orbán László Orban.Laszlo@uni-mate.hu, Annus Tamás tamas.annus@ttk.elte.hu, Müller Dalma mulda@caesar.elte.hu

HG

Dear All,

I confirm that I agree to the change.

Best regards,
Gabor Harami

Az [Android Outlook](#) letöltése

From: dalma muller <dalma.muller2@gmail.com>
Sent: Wednesday, March 23, 2022 9:20:26 AM
To: Jezsó Bálint <jezso.balint@ttk.elte.hu>
Cc: György Ullaga <ullagagyuri@gmail.com>; Varga Máté <mvarga@ttk.elte.hu>; Dr. Kovács Mihály <mihaly.kovacs@ttk.elte.hu>; Dr. Orbán László <Orban.Laszlo@uni-mate.hu>; Annus Tamás <tamas.annus@ttk.elte.hu>; Dr. Harami Gábor <gabor.harami@ttk.elte.hu>; Müller Dalma <mulda@caesar.elte.hu>
Subject: Re: Your confirmation needed for author change in our CDDIS-21-1343 ms

Dear all,

I confirm that I agree with this change

Best wishes,
Dalma Müller

Jezsó Bálint <jezso.balint@ttk.elte.hu> ezt írta (időpont: 2022. márc. 23., Sze, 14:04):

I agree with it.

Regards,
Bálint

Feladó: György Ullaga <ullagagyuri@gmail.com>
Elküldve: 2022. március 23., szerda 13:05
Címzett: Varga Máté <mvarga@ttk.elte.hu>
Másolatot kap: Dr. Kovács Mihály <mihaly.kovacs@ttk.elte.hu>; Dr. Orbán László <Orban.Laszlo@uni-mate.hu>; Jezsó Bálint <jezso.balint@ttk.elte.hu>; Annus Tamás <tamas.annus@ttk.elte.hu>; Dr. Harami Gábor <gabor.harami@ttk.elte.hu>; Müller Dalma <mulda@caesar.elte.hu>; dalma muller <dalma.muller2@gmail.com>
Tárgy: Re: Your confirmation needed for author change in our CDDIS-21-1343 ms

Dear all,

I confirm that I agree with this change

Best wishes,
György Ullaga

Máté Varga <mvarga@ttk.elte.hu> ezt írta (időpont: 2022. márc. 23., Sze, 12:55):

Dear all,

as I am sure you are all aware, after the first revision of our Blm manuscript we have included Barnabás Németh, a MSc student who did the bulk of the genotyping experiments on the authors' list. The CDDis Editorial Office however needs your written consent to this change.

So please reply ASAP to this email, using your email address that is also used in the CDDis manucript tracking system and confirm if you agree with this change.

Best regards

Best regards,
Máté

ELTE Eötvös Loránd University
Department of Genetics
Pázmány Péter stny. 1/C
H-1117, Budapest, Hungary

http://danio.elte.hu

From: **Dr. Orbán László** Orban.Laszlo@uni-mate.hu
Subject: Re: Your confirmation needed for author change in our CDDIS-21-1343 ms
Date: 23 March 2022 at 08:00
To: Varga Máté mvarga@ttk.elte.hu

OL

Dear Dr. Varga,

I am writing to inform you that I agree with the inclusion of Mr. Barnabás Németh into the authors' list of our Blm manuscript recently accepted for publication by CDD. The contribution of Mr Németh was essential for the production of the revised dataset that was accepted eventually by the journal.

Best regards:

László Orbán, PhD
Senior Research Advisor and Professor (adjunct)
Frontline Fish Genomics Research Group
Department of Applied Fish Biology
Institute of Aquaculture and Environmental Safety
The Georgikon Campus of Hungarian University of Agriculture and Life Sciences
Keszthely, Hungary

> On 2022. Mar 23., at 12:55, Máté Varga <mvarga@ttk.elte.hu> wrote:
>
> Dear all,
>
> as I am sure you are all aware, after the first revision of our Blm manuscript we have included Barnabás Németh, a MSc student who did the bulk of the genotyping experiments on the authors' list. The CDDis Editorial Office however needs your written consent to this change.
>
> So please reply ASAP to this email, using your email address that is also used in the CDDis manucript tracking system and confirm if you agree with this change.
>
> Best regards,
> Máté
>
> ---
> ELTE Eötvös Loránd University
> Department of Genetics
> Pázmány Péter stny. 1/C
> H-1117, Budapest, Hungary
>
> http://danio.elte.hu

From: **Dr. Kovács Mihály** mihaly.kovacs@ttk.elte.hu
Subject: RE: Your confirmation needed for author change in our CDDIS-21-1343 ms
Date: 23 March 2022 at 08:42
To: Varga Máté mvarga@ttk.elte.hu

KM

Dear All,

I agree with this change.

Mihaly Kovacs

Mihály Kovács, PhD

Professor
Head of Department of Biochemistry
ELTE-MTA "Momentum" Motor Enzymology Research Group

Eötvös Loránd University
H-1117 Budapest, Pázmány P. stny. 1/C, Hungary
Phone: +36-1-372-2500/8401
Fax: +36-1-381-2172
www.mk-lab.org
biokemia.elte.hu

From: Varga Máté <mvarga@ttk.elte.hu>
Sent: Wednesday, March 23, 2022 12:56 PM
To: Dr. Kovács Mihály <mihaly.kovacs@ttk.elte.hu>; "Dr. Orbán László" <Orban.Laszlo@uni-mate.hu>; Jezsó Bálint <jezso.balint@ttk.elte.hu>; Annus Tamás <tamas.annus@ttk.elte.hu>; Dr. Harami Gábor <gabor.harami@ttk.elte.hu>
Cc: György Ullaga <ullagagyuri@gmail.com>; Müller Dalma <mulda@caesar.elte.hu>; Dalma Meixner <meixnerdalma@gmail.com>
Subject: Your confirmation needed for author change in our CDDIS-21-1343 ms

Dear all,

as I am sure you are all aware, after the first revision of our Blm manuscript we have included Barnabás Németh, a MSc student who did the bulk of the genotyping experiments on the authors' list. The CDDis Editorial Office however needs your written consent to this change.

So please reply ASAP to this email, using your email address that is also used in the CDDis manucript tracking system and confirm if you agree with this change.

Best regards,
Máté

ELTE Eötvös Loránd University
Department of Genetics
Pázmány Péter stny. 1/C
H-1117, Budapest, Hungary

http://danio.elte.hu
